# Comparisons of Accelerometer Variables Training Monotony and Strain of Starters and Non-Starters: A Full-Season Study in Professional Soccer Players

**DOI:** 10.3390/ijerph17186547

**Published:** 2020-09-09

**Authors:** Hadi Nobari, Rafael Oliveira, Filipe Manuel Clemente, Jose Carmelo Adsuar, Jorge Pérez-Gómez, Jorge Carlos-Vivas, João Paulo Brito

**Affiliations:** 1 Department of Exercise Physiology, Faculty of Sport Sciences, University of Isfahan, Isfahan 81746-7344, Iran; 2 Sports Scientist, Sepahan Football Club, Isfahan 81887-78473, Iran; 3 Sports Science School of Rio Maior–Polytechnic Institute of Santarém, 2140-413 Rio Maior, Portugal; jbrito@esdrm.ipsantarem.pt; 4 Research Centre in Sport Sciences, Health Sciences and Human Development, 5001-801 Vila Real, Portugal; 5 Life Quality Research Centre, 2140-413 Rio Maior, Portugal; 6 Escola Superior Desporto e Lazer, Instituto Politécnico de Viana do Castelo, Rua Escola Industrial e Comercial de Nun’Álvares, 4900-347 Viana do Castelo, Portugal; filipe.clemente5@gmail.com; 7 HEME Research Group, Faculty of Sport Sciences, University of Extremadura, 10003 Cáceres, Spain; carmelo.adsuar@gmail.com (J.C.A.); jorgepg100@gmail.com (J.P.-G.); jorgecv@unex.es (J.C.-V.)

**Keywords:** acceleration, deceleration, in-season, non-starters, pre-season, soccer, starters, training monotony, training strain

## Abstract

The purpose of this study was two-fold: (1) to describe weekly average values for training monotony (TM) and training strain (TS) and their variations across the full soccer season, based on the number of accelerations and decelerations; (2) to analyze the differences between starter and non-starter players on weekly average TM and TS values for the pre-season and three in-season periods. In total, 21 professional soccer players were evaluated over 48 weeks during the full-season. The TM and TS were calculated based on the number of accelerations and decelerations at zone 1, zone 2 and zone 3, respectively. The results revealed that starters presented higher values compared to non-starters throughout the full season for all variables analyzed (all, *p* < 0.05). Generally, there were higher values in the pre-season. Specifically, accelerations at zones 1, 2 and 3 revealed moderate to very large significance of the starters compared to non-starters over the full-season. Decelerations at zone 1, 2 and 3 presented moderate to nearly optimally significant greater weekly averages for starters compared to non-starters during the full season. In conclusion, the TM and TS values were higher for starters compared to non-starters through the full-season, which confirms that the training session does not provide a sufficient load to non-starter soccer players during the full-season.

## 1. Introduction

In many sports, a full season is divided into phases that include the off-season, pre-season and in-season. The main phases are pre-season and in-season. Usually, pre-season improves the physical fitness of the players, while in-season promotes the maintenance of the capacities developed during pre-season [1,2].

In soccer science, there are several studies that focus on the maintenance of physical fitness during an entire competitive season [3] in order to assist coaches in training periodization and performance optimization, so as to avoid and/or reduce critical periods of decreased fitness [4]. In addition to the knowledge about the overall running demands of training sessions and matches, it is important to understand intense periods and the actions that occur (i.e., sprints, repeated sprints, accelerations and decelerations), as they have a substantial influence on the biomechanical and cardiometabolic demands experienced by players. The evidence indicates that an increasingly greater number of accelerations and decelerations is performed at higher standards, and this needs to be a consideration when designing training plans [5,6,7,8,9,10].

Moreover, in soccer there are differences in the first team squad because only 11 players can participate in a competitive match, and usually this is the day of the week with higher load [11]. This is a major aspect that determines different week loading patterns, depending on the regularity of a player starting a match or not. In this way, discrepancies in physical loads between players could lead to differences in important components of soccer-specific fitness, which may subsequently be presented on match day when players not accustomed to competitive match loads are required to complete the habitual physical loads performed by regular starting players. The challenge of maintaining squad physical fitness throughout the season is also technically difficult, given both organisational and traditional training practices inherent to professional soccer [12].

To better understand the differences between starter and non-starter soccer players, and to accomplish better training periodization and performance optimization strategies, training load should be monitored. For instance, quantifying external training loads has been extensively used and well discussed in sports, such as in soccer [13]. Through global positioning systems (GPS), there are different variables that can be assessed, such as total distance, different threshold speed distances covered, and acceleration and deceleration.

High-intensity activities (e.g., sprints, accelerations or decelerations) occur during decisive moments of soccer, such as contests for the ball, offensive or defensive actions, and goal-scoring opportunities [14,15], and may affect the match result. Therefore, coaches and researchers are constantly looking for better and more effective training methods to both improve and optimize the maximum acceleration capability of professional soccer players during linear sprinting and upon changes of direction speed. During the initial phases of sprinting, the maximum acceleration rate occurs when athletes increase their velocity [6,16,17].

However, soccer has also been reported to have a greater frequency of high and very high intensity decelerations compared to accelerations. Importantly, the damaging consequences of frequent and intense decelerations imply that specific loading strategies, to protect players from negative deceleration outcomes, may be advisable [9]. Intense accelerations and decelerations could make players particularly vulnerable to neuromuscular fatigue, and consequently to an exacerbated risk of incurring injury. Although accelerations and decelerations have substantial influence on external mechanical work [9,18], there are some discussions about the metric qualities of these variables, because they do not always provide valuable insights that give practical guidelines for training [8].

Thus, some studies provided more information when focusing on training monotony (TM: mean of training load during the seven days of the week divided by the standard deviation of the training load of the seven days) and training strain (TS: multiplication of accumulated weekly load by the TM) workload indices [19,20,21,22,23,24]. The knowledge of the variations in the load within and between weeks, and these variations’ relationships with the load distribution, is very useful to understand the impact of training strategies imposed by coaches and the physiological adaptations of the players [25].

There are few analyses that include workload indices, comparisons between starters and non-starters and different phases of the season simultaneously. In addition, to the authors’ knowledge, there is no exploration in the literature regarding workload indices produced through the metrics of acceleration and/or deceleration. Such information would be very practical, with a theoretical value for coaches and researchers, as it may help coaches with regard to the variations in acceleration and deceleration throughout the numerous training sessions over pre-season and in-season periods.

Therefore, the purpose of this study was two-fold: (1) to describe a weekly average for TM and TS values and their variations across the full season based on number of accelerations and decelerations; (2) to analyze the differences between starter and non-starter players on weekly average TM and TS values, based on number of accelerations and decelerations for the pre-season and in-season periods.

## 2. Materials and Methods

### 2.1. Experimental Approach to the Problem

The study included a full season of studying a professional football team for 48 weeks in the Persian Gulf Premier League and knockout tournament in the 2018–2019 year. The 48 weeks of the full season were divided into four periods (pre-season, W1 to W5; early-season, W6 to W19; mid-season, W20 to W34; and end-season, W35 to W48) to analyze the differences between starter and non-starters player on their weekly averages. Table 1 presents training, match and total time sessions for the different periods of the season.

This study monitored all the players’ speed activities (including training and competitions) throughout the season. The two aims of this study were as follows: (i) To describe mean/standard deviation (SD) weekly averages for TM and TS and their variations across the full season based on number of accelerations in zones 1 (AccZ1), 2 (AccZ2) and 3 (AccZ3), and the number of decelerations in zones 1 (DecZ1), 2 (DecZ2) and 3 (DecZ3). (ii) To analyze the differences between starter and non-starter players for the full season and during 4 periods of the season (pre-, early-, mid- and end-season) on weekly average TMAccZ1, TMAccZ2 and TMAccZ3, weekly average TSAccZ1, TSAccZ2 and TSAccZ3, weekly average TMDecZ1, TMDecZ2 and TMDecZ3, and weekly average TSDecZ1, TSDecZ2 and TSDecZ3.

### 2.2. Participants

In total, 21 professional soccer players (age, 28.3 ± 3.8 years; height, 181.2 ± 7.1 cm; body mass, 74.5 ± 7.7 kg; BMI, 22.6 ± 1.0 kg/m^2^) of one team competing in the Iranian Premier League were evaluated during 48 weeks of the full season. The inclusion criteria for the participants were as follows: (i) At least three training sessions per week. The exclusion criteria were as follows: (i) The lack of player information for two weeks in a row caused it to be excluded. (ii) To match the information in terms of physical activity, goalkeepers were excluded from the study.

The criteria to define starters and non-starters were assessed week by week according to a player’s attendance time at the match and training. Participants were divided into two groups, starters (*n* = 10) and non-starters (*n* = 11), considering the total playing time during the competition match of every week. To be considered as a starter, the player needed to complete at least 60 min of play during the competition.

Prior to the study, the club’s official license and head coach were obtained for research and it was done by the club coaches. Prior to commencing the study, we also received the approval of the research ethics committee from University of Isfahan. All players were informed of the purpose of the study, then signed the informed consent and also followed the Helsinki Declaration.

### 2.3. Monitoring External Load

#### 2.3.1. GPS Receiver Specifications

Full information on players’ training sessions and matches were collected by GPSports systems Pty Ltd. (Model: SPI High Performance Unit, Canberra, Australia). This GPS model’s features include the following—15 Hz position GPS, distance and speed measurement; accelerometer: 100 Hz, 16 G Tri-Axial-Track impacts, accelerations and decelerations as well as data source body load (BL); Mag: 50 Hz, Tri-Axial; dimensions: smallest device on the market (74 mm × 42 mm × 16 mm); robustness; SPI high performance unit based on Mining/Industrial Strength Electronics design; water resistance and data transmission: infra-red and weighs 56 g. Previously, it has been shown that the GPS unit, which was assayed as having a very high accuracy, showed validity and inter-unit reliability [26]. The data collected in terms of weather were in suitable GPS satellite conditions.

#### 2.3.2. Data Collection

In each training session, the GPS device was placed vertically in the belt bag. Then, before starting the warm-up, we made sure that the green and red lights are on for GPS tracking and heart-rate tracking, respectively. At the end of each training session, the GPS unit left the place. Then the entered the dock station for the device and the eventually to transfer data to automatically entered the computer by the AMS updated software. All data full season was set and collected by Default Zone in the SPI IQ Absolutes. The following variables were collected and analyzed: Duration (DR); Accelerations Zone1 (<2 m/s^2^) (AccZ1); Accelerations Zone2 (2 to 4 m/s^2^) (AccZ2); Accelerations Zone3 (>4 m/s^2^) (AccZ3); Decelerations Zone1 (<−2 m/s^2^) (DecZ1); Decelerations Zone2 (−2 to −4 m/s^2^) (DecZ2); Decelerations Zone3 (>−4 m/s^2^) (DecZ3) [27].

#### 2.3.3. Calculate Training Load

In this study, the weekly acute load (wAL) for each variable was determined for the total sessions, which was maintained per week. The following variables were obtained: (i) wAL, the accumulated daily number of variables during 1 week; (ii) weekly training monotony (wTM), the relation of wAL to SD during 1 week; (iii) weekly training strain (wTS), the multiplication of wAL by wTM. For other variables calculated by form, the weekly averages of all zone accelerations and decelerations for the in-season periods were used.

### 2.4. Statistical Analysis

Statistical procedures and computations were conducted using SPSS (version 25.0; IBM SPSS Inc., Chicago, IL, USA). Data are presented as mean and SD. Shapiro–Wilk and Levene’s tests were applied to check the normality and homogeneity of the data, respectively. Then, inferential tests were executed. Independent samples T-test were applied to analyze between group differences in all dependent derived GPS variables for the different periods of the season. Hedge’s *g* effect size (95% confidence interval) was also calculated. The Hopkins’ thresholds for effect size statistics were used, as follows: ≤0.2, trivial; >0.2, small; >0.6, moderate; >1.2, large; >2.0, very large; and >4.0, nearly perfect [28]. Differences were considered significant for *p* ≤ 0.05.

## 3. Results

**Figure 1:**
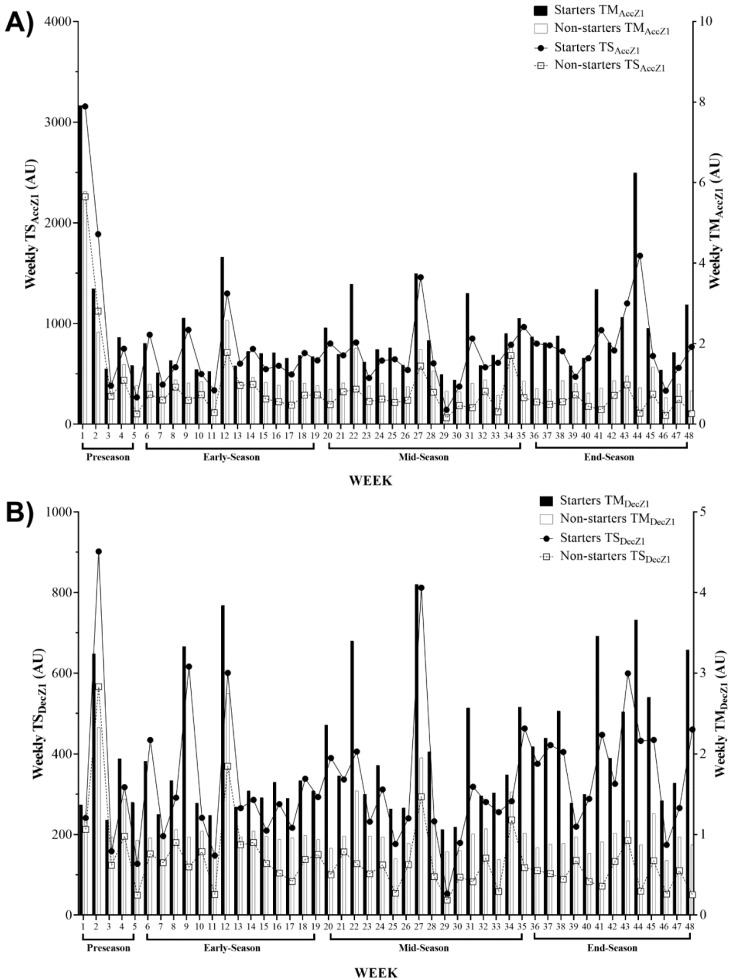
Descriptive statistics of weekly average for training monotony and training strain and their variations across the full season based on (**A**) number of accelerations in zone 1 (<2 m·s^−2^) and (**B**) number of decelerations in zone 1 (>−2 m·s^−2^). AU, arbitrary units; TM_AccZ1_, weekly training monotony based on number of accelerations in zone 1 (<2 m·s^−2^); TS_AccZ1_, weekly training strain based on number of accelerations in zone 1 (<2 m·s^−2^); TM_DecZ1_, weekly training monotony based on number of decelerations in zone 1 (>−2 m·s^−2^); TS_DecZ1_, weekly training strain based on number of decelerations in zone 1 (>−2 m·s^−2^).

Figure 1 shows an overall vision of the weekly average TM and TS variations, based on the number of accelerations (TM_AccZ1_ and TS_AccZ1_, respectively) and decelerations (TM_DecZ1_ and TS_DecZ1_, respectively) in the zone 1 data, across the full-season and its different periods (pre-season, early-season, mid-season and end-season) for starter and non-starter players. Overall, the highest TM_AccZ1_ and TS_AccZ1_ occurred in week 1 for both starters and non-starters. The lowest TM_AccZ1_ happened in week 30 and week 46 for starters and non-starters, respectively, while both groups presented the lowest TS_AccZ1_ in week 29 (Figure 1A). Starters experienced the highest TM_DecZ1_ in week 27 and non-starters in week 12, while the lowest TM_DecZ1_ occurred in week 29 and week 46 for starters and non-starters, respectively. Furthermore, both groups presented the highest TS_DecZ1_ in week 2, and the lowest TS_DecZ1_ in week 29 (Figure 1B).

**Figure 2:**
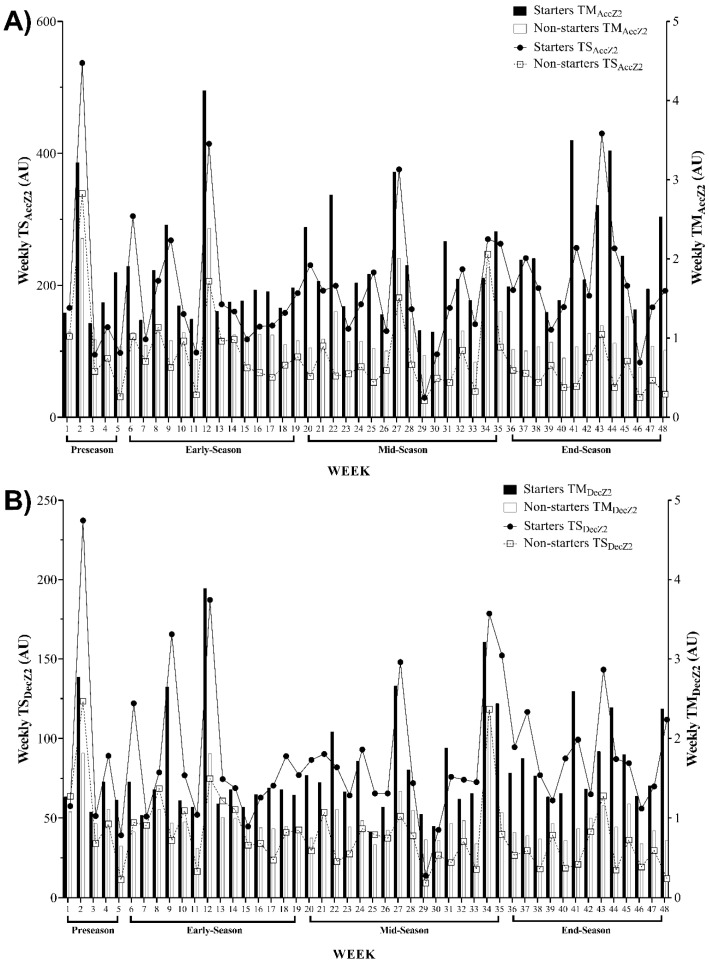
Descriptive statistics of weekly average for training monotony (TM) and training strain (TS) and their variations across the full season based on (**A**) number of accelerations in zone 2 (2 to 4 m·s^−2^) and (**B**) number of decelerations in zone 2 (−2 to −4 m·s^−2^). AU, arbitrary units; TM_AccZ2_, weekly training monotony based on number of accelerations in zone 2 (2 to 4 m·s^−2^); TS_AccZ2_, weekly training strain based on number of accelerations in zone 2 (2 to 4 m·s^−2^); TM_DecZ2_, weekly training monotony based on number of decelerations in zone 2 (−2 to −4 m·s^−2^); TS_DecZ2_, weekly training strain based on number of decelerations in zone 2 (−2 to −4 m·s^−2^).

Figure 2 illustrates an overall vision of the weekly average training monotony (TM) and training strain (TS) variations, based on the number of accelerations (TM_AccZ2_ and TS_AccZ2_) and decelerations (TM_DecZ2_ and TS_DecZ2_) in the zone 2 data, across the full season and its different periods for starter and non-starter players. Overall, the starters and non-starters presented the highest TM_AccZ2_ and TS_AccZ2_ values in weeks 12 and 2, respectively. The lowest TM_AccZ2_ happened in week 30 and week 46 for starters and non-starters, respectively. Coincidently, both groups experienced the lowest TS_AccZ2_ in week 29 (Figure 2A). Furthermore, starters presented the highest TM_DecZ2_ in week 12 and the lowest TM_DecZ2_ in week 25, while the highest and the lowest TM_DecZ2_ values were observed in week 34 and week 11 for non-starters, respectively. Coincidently, the highest and the lowest TS_DecZ2_ values arose in weeks 2 and 29, respectively, for both groups (Figure 2B). 

**Figure 3:**
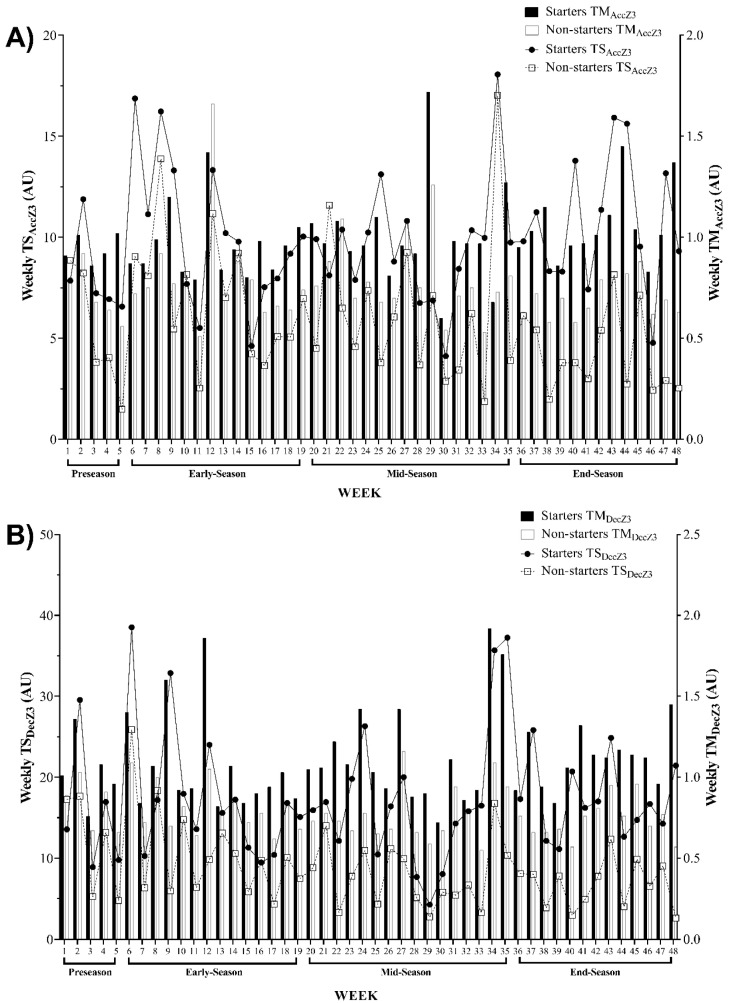
Descriptive statistics of weekly average for training monotony (TM) and training strain (TS) and their variations across the full season based on (**A**) number of accelerations in zone 3 (>4 m·s^−2^) and (**B**) number of decelerations in zone 3 (<−4 m·s^−2^). AU, arbitrary units; TM_AccZ3_, weekly training monotony based on number of accelerations in zone 3 (>4 m·s^−2^); TS_AccZ3_, weekly training strain based on number of accelerations in zone 3 (>4 m·s^−2^); TM_DecZ3_, weekly training monotony based on number of decelerations in zone 3 (<−4 m·s^−2^); TS_DecZ3_, weekly training strain based on number of decelerations in zone 3 (<−4 m·s^−2^).

Figure 3 displays an overall vision of the weekly average TM and TS variations, based on the number of accelerations (TM_AccZ3_ and TS_AccZ3_) and decelerations (TM_DecZ3_ and TS_DecZ3_) in the zone 3 data, across the full season and its different periods for starter and non-starter players. Overall, the highest TM_AccZ3_ values occurred in weeks 29 and 12, for starters and non-starters, respectively. Starters presented the lowest TM_AccZ3_ in week 30, while non-starters showed it in week 11. Coincidently, the highest TS_AccZ3_ values were observed in week 34 for both groups. However, starters presented the lowest TS_AccZ3_ in week 30 and non-starters showed it in week 5 (Figure 3A). Besides, non-starters showed the highest TM_DecZ3_ in week 27, while starters presented it in week 34. The lowest TM_DecZ3_ values occurred in week 30 and week 33 for starters and non-starters, respectively. Coincidently, both groups presented the highest TS_DecZ3_ in week 6, while the lowest TS_DecZ3_ was observed in week 29 for starters and week 48 for non-starters (Figure 3B).

Table 2 shows the between-group comparisons for weekly average TM_AccZ1_, TS_AccZ1_, TM_AccZ2_, TS_AccZ2_, TM_AccZ3_ and TS_AccZ3_ for the different periods of the season. Coincidently, the results revealed the moderately to very significantly greater weekly average TM_AccZ1_, TS_AccZ1_, TM_AccZ2_, TS_AccZ2_, TM_AccZ3_ and TS_AccZ3_ values of starters compared to non-starters during the pre-season (TM_AccZ1_: *p* = 0.015, *g* = −1.11; TS_AccZ1_: *p* = 0.013, *g* = −1.15; TM_AccZ2_: *p* < 0.001, *g* = 1.80; TS_AccZ2_: *p* = 0.001, *g* = −1.62; TM_AccZ3_: *p* < 0.001, *g* = −2.03; and TS_AccZ3_: *p* = 0.008, *g* = −1.24), early-season (TM_AccZ1_: *p* < 0.001, *g* = −2.67; TS_AccZ1_: *p* < 0.001, *g* = −2.87; TM_AccZ2_: *p* < 0.001, *g* = −2.43; TS_AccZ2_: *p* < 0.001, *g* = 2.07; TM_AccZ3_: *p* = 0.015, *g* = −1.12; and TS_AccZ3_: *p* = 0.024, *g* = −1.03), mid-season (TM_AccZ1_: *p* < 0.001, *g* = −2.86; TS_AccZ1_: *p* < 0.001, *g* = −2.99; TM_AccZ2_: *p* < 0.001, *g* = −2.70; TS_AccZ2_: *p* < 0.001, *g* = −2.65; TM_AccZ3_: *p* = 0.005, *g* = −1.33; and TS_AccZ3_: *p* = 0.001, *g* = −1.72) and end-season (TM_AccZ1_: *p* < 0.001, *g* = −2.62; TS_AccZ1_: *p* < 0.001, *g* = −2.82; TM_AccZ2_: *p* < 0.001, *g* = −2.92; TS_AccZ2_: *p* < 0.001, *g* = −3.54; TM_AccZ3_: *p* < 0.001, *g* = −2.82; and TS_AccZ3_: *p* < 0.001, *g* = −3.29).

Table 3 shows the between-group comparisons for weekly average TM_DecZ1_, TS_DecZ1_, TM_DecZ2_, TS_DecZ2_, TM_DecZ3_ and TS_DecZ3_ values for the different periods of the season. Similar to the outcomes obtained for parameters based on accelerations, moderate to nearly perfect significantly greater weekly average TM_DecZ1_, TS_DecZ1_, TM_DecZ2_, TS_DecZ2_, TM_DecZ3_ and TS_DecZ3_ values were derived from the starters compared to non-starters during the pre-season (TM_AccZ1_: *p* = 0.003, *g* = −1.43; TS_AccZ1_: *p* = 0.002, *g* = −1.52; TM_AccZ2_: *p* < 0.001, *g* = −1.78; TS_AccZ2_: *p* = 0.002, *g* = −1.55; TM_AccZ3_: *p* = 0.003, *g* = −1.48; and TS_AccZ3_: *p* = 0.048, *g* = −0.89), early-season (TM_AccZ1_: *p* < 0.001, *g* = −2.72; TS_AccZ1_: *p* < 0.001, *g* = −2.75; TM_AccZ2_: *p* < 0.001, *g* = −2.84; TS_AccZ2_: *p* < 0.001, *g* = −2.35; TM_AccZ3_: *p* < 0.001, *g* = −2.48; and TS_AccZ3_: *p* < 0.001, *g* = −1.84), mid-season (TM_AccZ1_: *p* < 0.001, *g* = −3.63; TS_AccZ1_: *p* < 0.001, *g* = −3.53; TM_AccZ2_: *p* < 0.001, *g* = −2.95; TS_AccZ2_: *p* < 0.001, *g* = −2.85; TM_AccZ3_: *p* < 0.001, *g* = −2.29; and TS_AccZ3_: *p* < 0.001, *g* = −2.20) and end-season (TM_AccZ1_: *p* < 0.001, *g* = −2.85; TS_AccZ1_: *p* < 0.001, *g* = −3.21; TM_AccZ2_: *p* < 0.001, *g* = −3.37; TS_AccZ2_: *p* < 0.001, *g* = −4.56; TM_AccZ3_: *p* < 0.001, *g* = −2.98; and TS_AccZ3_: *p* < 0.001, *g* = −3.33).

## 4. Discussion

The aims of this study were as follows: (1) to describe TM and TS and their variations across four periods of the season, based on the number of accelerations and decelerations; (2) to analyze the differences between starter and non-starter players in terms of TM and TS based on the number of accelerations and decelerations across four periods of the season. The first aim was accomplished and can be observed in Figure 1, Figure 2 and Figure 3. Moreover, as expected, a major finding revealed a meaningful variation in the workload indices of starters and non-starters.

Regarding the variations described in Figure 1, Figure 2 and Figure 3, there are some coincident findings that should be highlighted. The highest TM_AccZ1_ and TS_AccZ1_ occurred in week 1 for both starters and non-starters. These results were expected for the first week of training sessions (pre-season), whereat a possible higher training load was applied. Although it was not a purpose of this study to compare different periods of the season, the results are in line with some studies [19,20,29], which means that the exercise training program, early in the pre-season, focused on improving physical condition through a higher training load [30]. 

Then, a relevant variation occurred in the second week, wherein the TS_DecZ1_ and TS_AccZ2_ values were revealed to be the highest through the full-season. On week 6, the highest TS_DecZ3_ occurred for both starters and non-starters_._ A “w-shape” remained until week 12, when the highest TM_AccZ2_ occurred for starters and non-starters, and the highest TM_DecZ2_ for starters and the highest TM_AccZ3_ for non-starter occurred. Moreover, a “w-shape” remained until week 27, whereat the starters experienced the highest TM_DecZ1_ and non-starters showed the highest TM_DecZ3_. Then, on week 29, the lowest TS_AccZ1_ and the lowest TS_DecZ1_ occurred for starters and non-starters. The lowest TM_DecZ1_ also occurred for starters in the same week. Then, the lowest TS_DecZ2_ and TS_AccZ2_ for both starters and non-starters, and the lowest TS_DecZ3_ for starters, occurred in week 29 for all. On week 29, the lowest TM_AccZ3_ occurred, and on week 30 the lowest TM_AccZ1_ and TM_DecZ3_ occurred for starters. In this week, TM and TS showed a higher tendency towards lower loads for non-starter players, which could be associated with the end of the season and the importance given to starter players when compared to non-starter players. 

While for the beginning of the season (pre-season) it is easy to explain the results via the similarities between starters and non-starters in the weeks with higher TM and TS, it is not clear why there is an overall “w-shape” through the season. There are some contextual variables, such as match location, match result, quality of the opponent, tactic system and exercise training program applied, which could explain the data. Contextual factors, such as tactical formation, strength of opposition and match stoppages, may influence overall workloads during matches, and consequently have an impact on the previous or next training sessions. The evidence indicates that players competing in some match formations (3–5–2) could cover more total distance and perform higher-speed running, as well as performing more accelerations/decelerations compared to other formations [31].

In fact, few studies used this approach of calculating TM and TS [19,20,21,23]. Lazarus et al. [23] found that TM and TS revealed trivial effects on Australian soccer training performance. The same authors showed that the variations in those indices were difficult to understand, as they were in the present study. Delecroix et al. [21] showed that a regular workload is an injury-protective factor, while high TS is a risk factor when it is sustained for four weeks. As mentioned before, our study presented a “w-shape” that could have possible negative effects on the players, but injuries were not analyzed. In opposition to the studies of Clemente et al. [19,20], where TM showed a tendency to decrease as the weeks progressed, the present study did not present the same pattern. Indeed, there were variations throughout the full season. However, it is important to highlight that the present study used acceleration and deceleration to calculate TM and TS, and the previous studies mentioned [19,20,21,23] used session rate of perceived exertion (s-RPE). 

Although all comparisons between starters and non-starters in the various acceleration and deceleration thresholds are statistically different, the objective for this team’s pre-season training may have been to establish a chronic load, which was reduced in the early-season and in other periods throughout the competitive season. It is likely that this reduction in strain in some periods of the season was a deliberate attempt to reduce training volume, provide adequate recovery and maintain fitness and freshness. There are several contextual factors that could also influence this reduction in training load (training strain) during some portions of the season (e.g., congested match schedule, playoffs, and increased injury rate).

With respect to the second aim of the study, it is important to acknowledge that the soccer game has developed in the last few years. Players have more tactical responsibilities whilst in and out of possession and during ball possession transitions [32]. With such roles, players must be able to perform frequent intense acceleration and deceleration actions. High-intensity accelerations and decelerations are two very important metrics of external load. Both make distinctive and disparate internal physiological and mechanical loading demands on players [33]. On one hand, a higher metabolic cost emerges with acceleration [34]. On the other hand, a higher mechanical load manifests with decelerations [7] through the high impact peaks, loading rates [35] and possibly higher damage on soft-tissue structures [36]. This is why the frequency of accelerations and decelerations is associated with reductions in neuromuscular performance after the matches [37]. Even with the non-positive effects presented, elite players can perform a higher number of accelerations and decelerations than the lower-level players [38]. This statement can help to understand the differences between starters and non-starters in the present study. Overall, starters presented significatively higher values of TM and TS through the four periods of the season. This finding reinforces that the training load applied to non-starter players was not enough to produce more adaptations and to ensure players evolved as the weeks progressed. Coaches and their staff need to adjust their exercise training programs in order to develop and to apply similar loads to non-starter players.

Although Anderson et al. [12] reported that in the English Premier League, non-starters performed significantly less running (14.4–19.8 km/h), high speed running (19.9–25.1 km/h) and sprinting (>25.2 km/h) than starter players, in the present study, the analysis of movements of high demand and neuromuscular wear, such as accelerations and decelerations, showed significant differences between both groups. The results are indicators that monitoring training load must be carried out while considering the different metrics available to coaches and technical teams.

Some limitations should be addressed. The small sample size regarding number of players and teams analyzed constitute a limitation in the present study, but these issues are frequent in longitudinal studies over a full season in professional contexts, as reported by Clemente et al. [19]. Furthermore, differences in player positions were not analyzed, and this could influence the data analysis. There could also possibly exist different results if they were analyzed. For instance, the study of Clemente et al. [19] found small-to-moderate effect size differences for the number of sprints in acute load, TM and TS through different external load metrics. Moreover, the present study does not consider individual differences in acceleration and deceleration capacities, which can result in different results [5].

It is relevant to mention that when accelerometers are worn on the upper body, as they were in the present study, the crania–caudal axis of the accelerometer will likely only be close to equivalent with the global vertical axis when standing up-right or performing movements in the vertical plane. Any deviation from the assumed vertical orientation of the device may influence the accelerometer’s accuracy. 

Finally, as reported in the Clemente et al. [19] study, internal load, s-RPE, was not used and usually this variable is used to calculate training monotony and training strain which was not the purpose of the present study. Finally, this study did not consider the results of the match that may affect the collective behavior and, naturally, the demands imposed on players. Further research should analyze the impact of the matches as well as the competitive level of the team in analyses of workload.

Despite the limitations mentioned, this study was one of the first to analyze the variations in TM and TS between periods of the season, as well as between starters and non-starters through the acceleration and deceleration metrics. 

This study provides further knowledge regarding the variation profiling of acceleration and deceleration metrics during an entire soccer season. As it has been reported, a high frequency of rapid decelerations leaves players vulnerable to muscle damage and to chronic fatigue, which can lead to reductions in the performance of activities such as sprinting and changing direction [39]. Understanding how specific training sessions, match-play activities and contextual factors (e.g., formation model, play away or at home, opponent level) may influence player fatigue and recovery profiles is significantly relevant to practitioners/coaches.

The evident unpredictability of loads associated with decelerating rapidly also has important implications for the management of loads throughout the season; as suggested in Harper et al.’s [36] study, exercise training sessions should include specific exercises for offensive linesmen, which target the development of the neuromuscular capabilities required to produce and attenuate the high forces associated with decelerating rapidly or the braking forces during emergent and unpredictable situations.

Status differences should also be taken into account when planning and prescribing training loads across a full season.

## 5. Conclusions

Soccer involves a higher frequency and number of accelerations and decelerations, so training activities and match requirements must be managed throughout the microcycles by controlling the monotony and strain of the training load. This study gives new insights concerning the variation profiling of TS and TM, calculated through the accelerations and decelerations during the full season. It shows that there are significant differences between starters and non-starters in the four different periods analyzed during the full-season. Furthermore, there are some physiological adaptations that do not occur when players do not participate in matches. They correspond to the higher loads that are not reached on training sessions. Considering this information and the variability of the TM and TS presented, it is important for coaches, practitioners and scientists to monitor the demands imposed on their own group of players in order to better periodize and plan training sessions, and impose the proper load on starter and non-starter players throughout the full-season.

## Figures and Tables

**Table 1:** Training, match and total time sessions measured separately during the periods of the season.

Variables	Pre-Season	Early-Season	Mid-Season	End-Season
**Weeks (*n*)**	5	14	15	14
**Training Sessions (*n*)**	27	64	57	52
**Matches (*n*)**	1	15	15	13
Total Time * (min)	1805.4 ± 602.7	4636.7 ± 1314.2	4014.0 ± 1418.3	3670.6 ± 1353.2

* Total Time = The average of the total duration for every week + the whole weeks of that phase.

**Table 2:** Differences between starters and non-starters in terms of training monotony and training strain based on acceleration-derived GPS variables in the full season and its different periods.

TLP	Season Period	Group		%Difference (Non-Starters–Starters)	*p*	Hedge’s *g* (95% CI) (Non-Starters–Starters)
		Starters	Non-Starters			
**TM**_**AccZ1**_ **(AU)**	Pre-season	3.25 (0.62)	2.18 (1.14)	−41.0 (−60.6 to −11.8)	**0.015**	−1.11 (−2.03 to −0.19)
	Early-Season	1.87 (0.36)	1.13 (0.13)	−39.0 (−47.6 to −29.0)	**<0.001**	−2.67 (−3.85 to −1.49)
	Mid-Season	2.12 (0.37)	1.15 (0.27)	−46.1 (−55.1 to −35.3)	**<0.001**	−2.86 (−4.07 to −1.64)
	End-Season	2.48 (0.79)	0.98 (0.13)	−58.8 (−68.4 to −46.2)	**<0.001**	−2.62 (−3.79 to −1.45)
**TS**_**AccZ1**_ **(AU)**	Pre-season	1288.97 (265.15)	765.44 (547.51)	−56.7 (−78.0 to −15.1)	**0.013**	−1.15 (−2.07 to −0.23)
	Early-Season	659.98 (158.04)	306.50 (63.24)	−53.4 (−62.6 to −42.0)	**<0.001**	−2.87 (−4.09 to −1.65)
	Mid-Season	683.62 (153.10)	282.55 (101.83)	−59.9 (−69.2 to −47.7)	**<0.001**	−2.99 (−4.24 to −1.75)
	End-Season	793.44 (281.32)	216.63 (47.49)	−71.4 (−79.2 to −60.7)	**<0.001**	−2.82 (−4.02 to −1.61)
**TM**_**AccZ2**_ **(AU)**	Pre-season	1.82 (0.26)	1.19 (0.39)	−37.1 (−50.9 to −19.5)	**<0.001**	−1.80 (−2.81 to −0.78)
	Early-Season	1.76 (0.36)	1.09 (0.12)	−37.4 (−46.6 to −26.6)	**<0.001**	−2.43 (−3.56 to −1.30)
	Mid-Season	1.87 (0.27)	1.11 (0.27)	−41.5 (−50.6 to −30.8)	**<0.001**	−2.70 (−3.88 to −1.51)
	End-Season	2.10 (0.55)	0.93 (0.10)	−54.4 (−63.3 to −43.2)	**<0.001**	−2.92 (−4.15 to −1.69)
**TS**_**AccZ2**_ **(AU)**	Pre-season	209.71 (38.04)	112.24 (71.12)	−58.0 (−76.1 to −26.0)	**0.001**	−1.62 (−2.60 to −0.63)
	Early-Season	187.65 (55.12)	97.81 (23.62)	−47.4 (−59.6 to −31.5)	**<0.001**	−2.07 (−3.13 to −1.01)
	Mid-Season	188.62 (41.45)	87.19 (31.90)	−55.5 (−66.1 to −41.6)	**<0.001**	−2.65 (−3.82 to −1.48)
	End-Season	207.53 (54.59)	64.75 (12.88)	−68.3 (−75.1 to −59.7)	**<0.001**	−3.54 (−4.91 to −2.17)
**TM**_**AccZ3**_ **(AU)**	Pre-season	0.94 (0.10)	0.70 (0.13)	−26.5 (−36.0 to −15.7)	**<0.001**	−2.03 (−3.09 to −0.98)
	Early-Season	0.95 (0.14)	0.80 (0.11)	−15.4 (−25.5 to −4.0)	**0.015**	−1.12 (−2.05 to −0.20)
	Mid-Season	0.98 (0.09)	0.78 (0.18)	−21.8 (−32.4 to −9.5)	**0.005**	−1.33 (−2.28 to −0.39)
	End-Season	1.05 (0.14)	0.70 (0.09)	−33.1 (−40.7 to −24.5)	**<0.001**	−2.82 (−4.03 to −1.61)
**TS**_**AccZ3**_ **(AU)**	Pre-season	8.09 (2.40)	4.86 (2.59)	−46.8 (−65.6 to −17.6)	**0.008**	−1.24 (−2.18 to −0.31)
	Early-Season	10.17 (3.14)	7.24 (2.31)	−29.7 (−47.6 to −5.7)	**0.024**	−1.03 (−1.94 to −0.12)
	Mid-Season	9.65 (1.60)	6.12 (2.25)	−39.5 (−53.2 to −21.9)	**0.001**	−1.72 (−2.72 to −0.72)
	End-Season	10.61 (2.31)	4.35 (1.23)	−59.9 (−68.7 to −48.7)	**<0.001**	−3.29 (−4.61 to −1.98)

**Abbreviations:** TLP, training load parameters; AU, arbitrary units; TM_AccZ1_, weekly average training monotony based on number of accelerations in zone 1 (<2 m·s^−2^); TS_AccZ1_, weekly average training strain based on number of accelerations in zone 1 (<2 m·s^−2^); TM_AccZ2_, weekly average training monotony based on number of accelerations in zone 2 (2 to 4 m·s^−2^); TS_AccZ2_, weekly average training strain based on number of accelerations in zone 2 (2 to 4 m·s^−2^); TM_AccZ3_, weekly average training monotony based on number of accelerations in zone 3 (>4 m·s^−2^); TS_AccZ3_, weekly average training strain based on number of accelerations in zone 3 (>4 m·s^−2^); *p*, *p*-value at alpha level 0.05; Hedge’s *g* (95% CI), Hedge’s *g* effect size magnitude with 95% confidence interval. Significant differences (*p* ≤ 0.05) are highlighted in bold.

**Table 3:** Differences between starters and non-starters in terms of training monotony and training strain based on deceleration-derived GPS variables in the full-season and its different periods.

TLP	Season Period	Group		%Difference	*p*	Cohen’s *d* (95% CI)
		Starters	Non-Starters			
**TM**_**DecZ1**_ **(AU)**	Pre-season	1.82 (0.19)	1.31 (0.44)	−32.3 (−48.1 to −11.7)	**0.003**	−1.43 (−2.39 to −0.47)
	Early-Season	1.80 (0.33)	1.09 (0.16)	−39.3 (−48.2 to −29.0)	**<0.001**	−2.72 (−3.91 to −1.53)
	Mid-Season	1.98 (0.27)	1.06 (0.22)	−46.9 (−54.6 to −38.0)	**<0.001**	−3.63 (−5.02 to −2.24)
	End-Season	2.33 (0.67)	0.94 (0.12)	−58.2 (−67.3 to −46.5)	**<0.001**	−2.85 (−4.06 to −1.63)
**TS**_**DecZ1**_ **(AU)**	Pre-season	349.37 (42.58)	208.15 (116.22)	−52.0 (−72.0 to −17.8)	**0.002**	−1.52 (−2.49 to −0.55)
	Early-Season	315.25 (74.37)	151.32 (35.13)	−52.1 (−62.3 to −39.2)	**<0.001**	−2.75 (−3.95 to −1.56)
	Mid-Season	310.75 (62.22)	122.72 (38.47)	−61.6 (−69.9 to −50.9)	**<0.001**	−3.53 (−4.90 to −2.16)
	End-Season	373.32 (114.88)	103.09 (22.17)	−71.5 (−78.5 to −62.3)	**<0.001**	−3.21 (−4.51 to −1.92)
**TM**_**DecZ2**_ **(AU)**	Pre-season	1.56 (0.19)	1.07 (0.32)	−34.1 (−47.7 to −17.1)	**<0.001**	−1.78 (−2.79 to −0.77)
	Early-Season	1.56 (0.26)	0.97 (0.11)	−37.2 (−45.5 to −27.7)	**<0.001**	−2.84 (−4.06 to −1.63)
	Mid-Season	1.65 (0.24)	1.01 (0.17)	−38.9 (−47.0 to −29.5)	**<0.001**	−2.95 (−4.19 to −1.72)
	End-Season	1.73 (0.33)	0.88 (0.12)	−48.8 (−56.7 to −39.5)	**<0.001**	−3.37 (−4.70 to −2.04)
**TS**_**DecZ2**_ **(AU)**	Pre-season	94.91 (24.53)	50.88 (29.47)	−54.9 (−72.8 to −25.2)	**0.002**	−1.55 (−2.53 to −0.58)
	Early-Season	87.25 (20.56)	45.25 (13.33)	−48.9 (−60.9 to −33.3)	**<0.001**	−2.35 (−3.47 to −1.24)
	Mid-Season	86.08 (18.99)	38.33 (12.96)	−56.9 (−67.1 to −43.5)	**<0.001**	−2.85 (−4.06 to −1.63)
	End-Season	89.15 (28.96)	28.96 (6.29)	−67.6 (−73.7 to −60.1)	**<0.001**	−4.56 (−6.18 to −2.94)
**TM**_**DecZ3**_ **(AU)**	Pre-season	1.03 (0.11)	0.80 (0.18)	−23.9 (−35.8 to −9.7)	**0.003**	−1.48 (−2.44 to −0.51)
	Early-Season	1.08 (0.09)	0.81 (0.12)	−25.4 (−32.8 to −17.2)	**<0.001**	−2.48 (−3.61 to −1.34)
	Mid-Season	1.14 (0.19)	0.78 (0.11)	−31.4 (−40.4 to −21.1)	**<0.001**	−2.29 (−3.39 to −1.19)
	End-Season	1.11 (0.16)	0.74 (0.07)	−32.7 (−39.8 to −24.7)	**<0.001**	−2.98 (−4.23 to −1.74)
**TS**_**DecZ3**_ **(AU)**	Pre-season	15.76 (4.63)	10.77 (5.99)	−40.0 (−61.9 to −5.6)	**0.048**	−0.89 (−1.79 to 0.01)
	Early-Season	17.88 (3.47)	10.64 (4.03)	−43.4 (−57.5 to −24.8)	**<0.001**	−1.84 (−2.86 to −0.82)
	Mid-Season	17.36 (5.33)	7.98 (2.54)	−54.3 (−66.0 to −38.5)	**<0.001**	−2.20 (−3.28 to −1.11)
	End-Season	17.38 (4.22)	6.77 (1.30)	−60.7 (−68.4 to −51.0)	**<0.001**	−3.33 (−4.66 to −2.01)

**Abbreviations:** TLP, training load parameters; AU, arbitrary units; TM_DecZ1_, weekly average training monotony based on number of decelerations in zone 1 (> −2 m·s^−2^); TS_DecZ1_, weekly average training strain based on number of deceleration in zone 1 (> −2 m·s^−2^); TM_DecZ2_, weekly average training monotony based on number of decelerations in zone 2 (−2 to −4 m·s^−2^); TS_AccZ2_, weekly average training strain based on number of accelerations in zone 2 (2 to 4 m·s^−2^); TM_DecZ3_, weekly average training monotony based on number of decelerations in zone 3 (< −4 m·s^−2^); TS_DecZ3_, weekly average training strain based on number of decelerations in zone 3 (< −4 m·s^−2^); *p*, *p*-value at alpha level 0.05; Hedge’s *g* (95% CI), Hedge’s *g* effect size magnitude with 95% confidence interval. Significant differences (*p* ≤ 0.05) are highlighted in bold.
